# Heterogeneities of the perceptual-motor style during locomotion at height

**DOI:** 10.3389/fnhum.2023.1228195

**Published:** 2024-01-12

**Authors:** Danping Wang, Ioannis Bargiotas, Jiuwen Cao, Nicolas Vayatis, Laurent Oudre, Pierre-Paul Vidal

**Affiliations:** ^1^Plateforme d’Etude Sensorimotricité, Université Paris Cité, Paris, France; ^2^Machine Learning and I-Health International Cooperation Base of Zhejiang Province, Hangzhou Dianzi University, Hangzhou, China; ^3^Centre Borelli, CNRS, SSA, INSERM, Université Paris Cité, Université Paris Saclay, ENS Paris Saclay, Paris, France; ^4^Centre Borelli, CNRS, SSA, INSERM, Université Paris Saclay, Université Paris Cité, ENS Paris Saclay, Gif-sur-Yvette, France

**Keywords:** motor style, sensorimotor, locomotion, virtual reality, perceptive

## Abstract

In a recent review, we summarized the characteristics of perceptual-motor style in humans. Style can vary from individual to individual, task to task and pathology to pathology, as sensorimotor transformations demonstrate considerable adaptability and plasticity. Although the behavioral evidence for individual styles is substantial, much remains to be done to understand the neural and mechanical substrates of inter-individual differences in sensorimotor performance. In this study, we aimed to investigate the modulation of perceptual-motor style during locomotion at height in 16 persons with no history of fear of heights or acrophobia. We used an inexpensive virtual reality (VR) video game. In this VR game, Richie’s Plank, the person progresses on a narrow plank placed between two buildings at the height of the 30th floor. Our first finding was that the static markers (head, trunk and limb configurations relative to the gravitational vertical) and some dynamic markers (jerk, root mean square, sample entropy and two-thirds power law at head, trunk and limb level) we had previously identified to define perceptual motor style during locomotion could account for fear modulation during VR play. Our second surprising result was the heterogeneity of this modulation in the 16 young, healthy individuals exposed to moving at a height. Finally, 56% of participants showed a persistent change in at least one variable of their skeletal configuration and 61% in one variable of their dynamic control during ground locomotion after exposure to height.

## 1 Introduction

In a recent review (Mantilla et al., 2020), we summarized the characteristics of perceptual-motor styles in humans. Style can vary from individual to individual, task to task, and pathology to pathology, as sensorimotor transformations demonstrate considerable adaptability and plasticity. Although the behavioral evidence for individual styles is substantial, much remains to be done to understand the neural and mechanical substrates of inter-individual differences in sensorimotor performance. Perceptual-motor style may change during intensive physical activity or during the course of an illness, but this is no guarantee that it will be to the benefit of the individual, the athlete, or the patient. Numerous studies also show that perceptual-motor styles can evolve with proactive learning. So, whether training athletes, patients, or soldiers, the problem would be similar: identifying and longitudinally monitoring a person’s perceptual-motor style would help considerably in revealing the onset of a pathological process. Ultimately, this would make it possible to personalize training and/or treatment and to decide when they need to be readjusted to maintain optimal motor control.

To track a person’s perceptual-motor style, reliable markers of motor behavior need to be identified. To answer this question, we quantified motor behavior at rest, during walking, and during running at maximum speed (Vidal and Lacquaniti, 2021). We verified that motor control could be conveniently decomposed into static (stable head, trunk, and limb configurations relative to the gravitational vertical) and dynamic [jerks, root mean square (RMS), sample entropy, and the two-thirds power law quantifying head, trunk, and limb movements] components. We then postulated that markers identifying low intra-individual variability and high inter-individual variability were adequate to define the perceptual-motor style of individuals who otherwise exhibited high inter-individual variability.

Once these factors have been defined, one can tackle the question of how cognitive factors modulate motor perceptual style (Lelard et al., 2019; Williams et al., 2020). In the present study, we investigated locomotion during exposure to height. Studies have described three types of responses to height exposure (Brandt and Huppert, 2014; Brandt et al., 2015). The first response is a physiological height imbalance resulting from impaired visual control of balance: the distance from the stationary visual scene becomes too great to detect and counteract body movements. As a result, visual cues come into conflict with vestibular and proprioceptive cues. In addition, changes in attention can lead to threat-related postural changes (Huffman et al., 2009). In the second response, visual height intolerance induces a more or less pronounced apprehension of losing balance or falling. In the third response, acrophobia presents the same symptoms with such intensity that it can be considered a specific phobia, leading to panic attacks. Several types of symptoms can be observed during height exposure: anxiety, weak knees, and inner restlessness. Neurovegetative symptoms (accelerated heart rate, sweating, drowsiness, and tremors) are predominant. At the motor level, static postural control is impaired due to the co-contraction of the antigravity muscles, which stiffens the whole body. Oculomotor and head movements in all three dimensions are reduced and consist mainly of gaze fixation on the horizon. Individuals tend to walk slowly, stride length is reduced, and double-support phases are increased. All these symptoms are reinforced by anxiety and increased height. Postural symptoms saturate at approximately 20 m above ground and anxiety at approximately 40 m in non-acrophobic patients and 70 m in acrophobic participants (Teggi et al., 2019; Huppert et al., 2020).

Most studies of the threats posed by height to human postural control have focused on static balance. When locomotion was studied, the number of markers was limited. Moreover, these parameters were averaged across participants (see Section 4). This is why we have undertaken a quantitative analysis of perceptual-motor style and its inter-individual heterogeneity during height locomotion. To answer this question, we used the markers we had identified to characterize perceptual-motor style during locomotion at ground level (Vidal and Lacquaniti, 2021). The study involved 16 young individuals with no history of fear of heights or acrophobia. For height exposure, we used an inexpensive virtual reality (VR) video game, with the idea of later using the same protocol clinically to study how emotion modulates perceptual-motor style, including acrophobia and fear of falling. In this VR game, Richie’s Plank, the player must progress on a narrow plank placed between two buildings at the height of the 30th floor.

## 2 Materials and methods

### 2.1 Participants

We included 16 volunteers (12 men), with a mean age of 24 ± 2 years, 64 ± 16 kg, and 176 ± 6 cm tall. The data of the sixteen volunteers are available. Their body mass index corresponded to a normal range (*WHO | The World Health Report 2006—Working together for health*). *A priori* approval was obtained from the university’s research board (IRB CER no. 2021-12-WANGVIDAL, 6 April 2021), and written informed consent was obtained from all participants. Before the experiment, all participants were asked whether they had blood pressure or heart problems, whether they participated in regular physical activity, and whether they were afraid of heights. We excluded all participants with agoraphobia, acrophobia, and fear of heights.

### 2.2 Experimental protocol

All data collection took place at the Plateforme d’Etude de la Sensorimotricité at the Université de Paris Cité, Paris. We used a headset, the HTC Vive, to immerse the subjects in VR. To increase the anxiety level during locomotion, we used the Richie’s Plank video game: the person progressed on the ground and on a narrow plank placed between two buildings at the height of the 30th floor.

**Figure 1:**
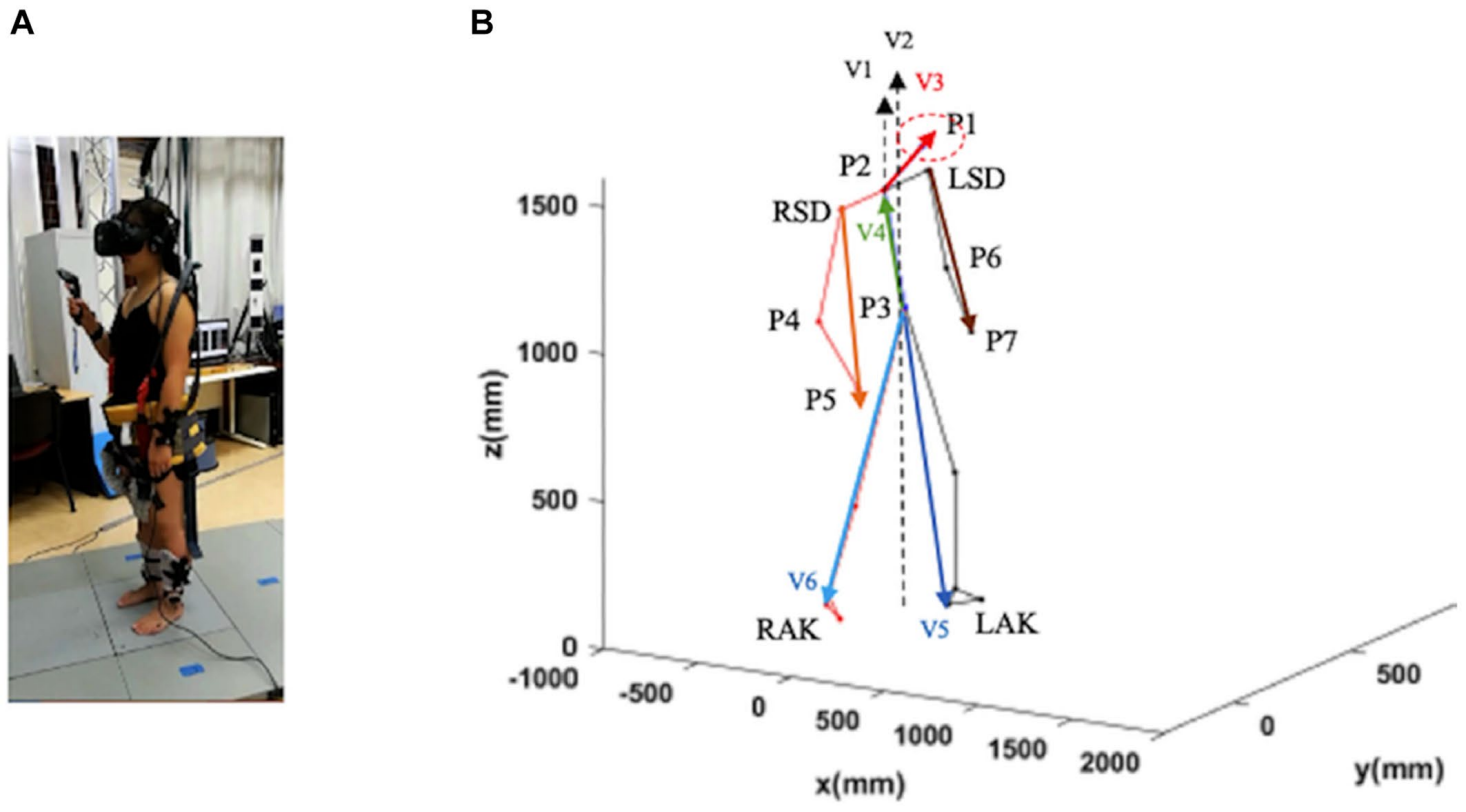
Schematic representation of a subject in a static position. Trunk inclination was defined as the angle between the gravity vector passing through P3 and the vector P3–P2 (V4; green vector). Head inclination was defined as the angle between the gravity through P2 and the vector P2–P1 (V3; red vector). Leg inclination (left and right) was defined as the angle between the gravity vector passing through P3 and the vector P3–LAK (V5; blue vector). and the vector P3–RAK (V6; blue vector). Arm inclination (left and right) was defined as the angle between the gravity vector passing through P2 and the vector LSD-P7 and the vector RSD-P5. LSD, left acromion; LAK, left ankle; P1–P7, virtual points; RSD, right acromion; RAK, right heel (see text for further details).

The recordings of the locomotion episodes were performed with the Codamotion 3D Analysis System (Charnwood Dynamics, Leicestershire, United Kingdom) (Figure 1A). Infrared light signals generated by the markers placed on the anatomical landmarks were captured by the Coda sensor module at 100 Hz. The data were processed with the Codamotion ODIN software on a personal computer with a Microsoft Windows-based operating system. A total of 24 Coda active markers were placed on the body in four segments (head, trunk, legs, and feet), with a minimum of three markers placed for each segment (Figure 1A). Markers were detected by four Coda CX1 units placed in the working space in the laboratory to cover the running range.

- Four markers were placed on the headset (one on the forehead was placed on the Xsens sensor, one each on the left temporal bone and right temporal bone, and one on the external occipital protuberance).
- Two markers were placed at the level of the left and right acromion.
- Two markers were placed at the left and right wrists and one marker at the left and right elbows.
- Two markers were placed at the level of the left and right hips.
- Two 4-marker clusters were installed below the right and left lateral condyles of the tibia.
- Six markers were placed on the feet (one marker each on the left and right heel elbow, on the left and right heel at the low level, and on the right and left fifth metatarsal-phalangeal joint).

The participant wearing the headset displaying the Richie’s Plank video game was barefoot and protected with a harness when walking. The protocol consisted of three scenarios.

1. The VR game started: the individual was in the elevator. The person decided it was time to start walking on the ground (G1 from now on) in a virtual landscape depicting a street. They were then asked to “stop and come back after 4,000 mm of progression.”
2. The individual was in the elevator and selected the “plank” with a VR handle. The elevator door opened when the subject “arrived” at the 30th floor (H from now on). A 200 × 4,000 mm virtual suspended board was in front of the subject. The subject was asked to walk on the suspended board and decided for himself when to start walking. The subject was asked to stop and then return to the elevator when he reached the end of the board. Then, the subject selected “ground” with a VR handle in the elevator and descended to ground level.
3. Scenario 1 was repeated (G2 from now on).

### 2.3 Data processing

#### 2.3.1 Static marker extraction

We defined seven virtual points based on the collected location data of markers for each part (P) (Figure 1B): P1: the midpoint of the four sensors on the head; P2: the midpoint of the line connecting the two shoulders; P3: the midpoint of the line connecting the two waist points; P4 and P6: the midpoint of the two sensors on the elbows; P5 and P7: the midpoint of the two sensors on the wrists.

To determine the skeletal configuration of each participant in the sagittal, frontal, and transversal planes, we computed four inclination angles:

1. Head inclination angle was calculated as the angle between the vector of P2-P1 (V2) and the vertical axis in the sagittal and frontal planes and the angle between the vector of P2-P1 (V2) and the anterior–posterior axis in the transverse plane.
2. Trunk inclination: the angle between the vector of P3-P2 and the vertical axis (V2) calculated in the sagittal and frontal planes, and the angle between the vector of P3-P2 (V4) and the anterior–posterior axis calculated in the cross-sectional plane.
3. Leg inclination: the angle between the vector of P3-left ankle (P3-LAK; V5) or P3-right ankle (P3-RAK; V6) and the vertical axis calculated in the sagittal plane and the frontal plane, and only the leg inclination of the forward step calculated. The angle between the vector of P3-LAK (V5) or P3-RAK (V6) and the anterior–posterior axis was calculated in the transversal plane.
4. Arm inclination: the angle between the vector right acromion-P4 (RSD-P5) or left acromion (LSD-P7) and the vertical axis V1 in the sagittal and frontal planes. The angle between the vectors RSD-P5 or LSD-P7 and the anterior–posterior axis was calculated in the transversal plane.

#### 2.3.2 Gait variable

We used six gait parameters. Of these, step width, step length, step time, and step height were calculated directly from the gait cycle using the markers mounted on each foot. We also calculated the velocity and acceleration of six body segments: head, trunk, arm, thigh, calf, and feet.

#### 2.3.3 Jerk

Jerk is the third derivative of position. It is a measure of the rate at which each part of the body is accelerated. First, the positional data for each marker were smoothed using a fourth-order, zero-lag Butterworth filter as described by Hreljac (1993). Second, the second derivative (acceleration) of the positional data involved using finite difference equations. Finally, the acceleration data were smoothed, and the first derivative of the acceleration $\frac{d\left(Acc\right)}{dt}$ (jerk) was calculated.

For the transversal plane, jerk was calculated as follows:


$$\mathrm{JERK}=\frac{1}{2}\overset{t}{\underset{0}{\int }}{\left(\frac{\mathrm{dAccX}}{dt}\right)}^{2}+{\left(\frac{\mathrm{dAccY}}{dt}\right)}^{2}$$

where *AccX* corresponds to the obtained acceleration in the medio-lateral axis and *AccY* corresponds to the obtained acceleration in the anterior–posterior axis. We calculate Jerk in the other planes by combining the acceleration in the other directions: *AccX* and *AccZ* for the frontal plane and *AccY* and *AccZ* for the sagittal plane.

#### 2.3.4 Root mean square (RMS)

The RMS of trunk acceleration is frequently used in gait analysis. For marker displacement, the RMS amplitude represents the standard deviation of the displacement of the marker. This parameter measures the average absolute displacement around the mean marker and is often used. For example, a decrease in the RMS amplitude of the center of pressure represents an increased ability to preserve an upright stance. An increased RMS value suggests a decreased ability to maintain postural control.

#### 2.3.5 Sample entropy

Sample entropy is a variation of the approximate entropy method. Sample entropy is the foundation for determining the complexity of both stationary and non-stationary signals. Sample entropy is the negative natural logarithm of the probability that two sequences will be similar for *m* + 1 data points divided by the probability that two sequences will be similar from data points. For two data points to match, they need to be within a range of tolerance of ± *r*, which is from 10% to 20% of the standard deviation of the original dataset. The match of the template sequence to itself is excluded from this calculation. The elimination of this self-matching is the distinction between sample entropy and approximate entropy. The equation for calculating the sample entropy of a set of time series data is as follows:


$$\mathrm{SampEnt}=\ln\left(\frac{\sum_{i=1}^{N-m}n_{i}^{\prime m}}{\sum_{i=1}^{N-m}n_{i}^{\prime m+1}}\right)$$

where *N* is the number of points in the dataset, $n_{i}^{\prime m}$ is the number of vector matches for vectors with length m, and $n_{i}^{\prime m+1}$ is the number of vector matches for vectors with length *m* + 1.

#### 2.3.6 Two-thirds power law

A mathematical equation known as the two-thirds power law, proposed by Lacquaniti et al. (1983), shows that the kinematics of many different human movements obey an identical relationship between the tangential velocity and the curvature of the motor trajectories. This law states that v = γκ^−β^ represents a robust local relationship between the geometrical and temporal aspects of human movement, represented by curvature κ and speed v, with a piecewise constant γ and exponent value β = ⅓. This law has been partially studied for the trajectory of the center of mass of the human body during walking (Tesio et al., 2011). The trajectory of the center of mass was segmented into high- and low-curvature segments. The β coefficient was close to the expected one-third value if the complete trajectory was considered. However, with high-curvature segments, the β coefficient is markedly higher (β = 0.486). By contrast, with low-curvature segments, the β coefficient is markedly lower (β = 0.185). In an equivalent form, let ω be the angular or curvilinear speed, r_c_ the radius of curvature, C = 1/r_c_ the curvature, and k a constant. In its simplest form, the law predicts that ω = kC^2/3^, where k is a constant. Hence, the popular term is the two-thirds power law.

### 2.4 Statistical analysis

Statistical analysis of individual gait and its dynamic parameters followed three different scenarios: 16 participants on ground (G1), aerial (H), and secondary ground (G2). For all comparisons, a *p*-value of ≤0.05 was considered statistically significant. Statistical analyses and variable computation involved using MATLAB 2019.

The current statistical procedure begins with the examination of multiple variables through a rigorous process. To assess whether there are statistically significant differences within each variable across our three conditions (G1, H, and G2), each variable underwent testing using a univariate repeated measures analysis of variance (rm-ANOVA), and their *p*-values were adjusted by Bonferroni correction (Table 1). This correction method divides the desired significance level (here a = 0.05) by the number of comparisons to ensure that the family-wise error rate is controlled at the desired level. If the univariate rm-ANOVA yields a significant result, indicating that there are indeed differences between the scenarios, a post-hoc analysis is conducted to delve deeper into the nature of these differences. We performed a typical post-hoc analysis, which involves pairwise comparisons through paired t-tests with Bonferroni adjustment.

**Table 1:** Outcomes of significance subsequent to the application of multiple univariate repeated measures analysis of variance (rm-ANOVA) with Bonferroni adjustment, indicated by a + sign.

	Head			Trunk			Arm			Thigh			Calf			Foot		
	Tr	Sg	Fr	Tr	Sg	Fr	Tr	Sg	Fr	Tr	Sg	Fr	Tr	Sg	Fr	Tr	Sg	Fr
ß factor										+	+		+					
Jerk																		
Entropy							+	+	+									
RMS																		
Angle																		
Speed	+			+			+			+			+			+		
Acc																		
	Step length			Step time			Step height			Step width								
Gait	+						+											

It is crucial to highlight that, for parameters retaining significance following the aforementioned (strong indeed) *p*-value adjustment, post-hoc pairwise analyses revealed significant differences across all pairs of conditions (G1, H, and G2).

Concerning the intra-individual differences in different scenarios, the variables that are computed per step (e.g., step length per step, step height per step, and angles per step) are examined through a systematic approach to maintain the overall Type I error rate while conducting multiple hypothesis tests.

In the first step of this procedure, an analysis of variance (ANOVA) is performed for each variable of interest, and the corresponding *p*-values are adjusted by Bonferroni.

For those variables that remain statistically significant, post-hoc analyses are conducted through pairwise t-tests. Importantly, the Bonferroni correction is once again applied at this stage, ensuring that the significance level for each pairwise comparison is appropriately adjusted to control the overall Type I error rate. By adhering to this two-step procedure, we can confidently explore multiple hypotheses without inflating the risk of Type I errors. The Bonferroni correction serves as a critical safeguard in the process, enabling rigorous statistical control throughout the multiple testing process, both in the initial ANOVA analyses and in subsequent post-hoc pairwise comparisons.

For those comparisons that remain significant after the above steps, the Cohen’s D effect size is calculated.

Cohen’s D categorizes the significant changes in three standard effect size classes: small (0.2 ≤ D < 0.5), medium (0.5 ≤ D < 0.8), and large (0.8 ≤ D). For each of these classes, we assign an arbitrary weight through the number of stars (*), * for small, ** for medium, and *** for large effect sizes, which will be used to weigh these differences appropriately.

Using the stars mentioned above, we finally define a score for each participant that indicates the overall statistical change that we were able to observe during locomotion at the level of the head, trunk, and limbs when comparing the characteristics during G1 vs. H, H vs. G2, and G1 vs. G2.

We have 16 types of parameters: Gait, 3D Angles, 3D ß factor, 3D Jerk, 3D RMS, and 3D entropy. For every individual, we checked for significant changes for every parameter ( p ), in every body segment ( bs ) in all three comparisons c (G1-H, H-G2, and G1-G2) in all three planes ( ax ). As was mentioned previously, every parameter was attributed by a number of stars s (no star for non-significant, * for small, ** for medium, and *** for large effect sizes).

Considering that the maximum number score ( MNS ) that someone can take per type is given by $MNS_{i}=p*bs*c*s*\mathrm{ax}$ , we calculate the 16 relative scores per individual as:


$$S_{i}=\frac{\mathrm{Scor}e_{i}}{MNS_{i}}$$

where $\mathrm{Scor}e_{i}$ is the summation of an individual’s stars per type. The final score $SC_{j}$ of every individual j is given by:

$$SC_{j}=\overset{21}{\underset{i=1}{\sum }}S_{i}$$

In this study, we considered six parameters for Gait in one plane, angles were calculated in four segments, and three planes and ß factors, Jerk, RMS, and entropy were calculated in six segments and three planes.

Let us give some examples of how the MNS is calculated:


$$\begin{array}{l} MNS_{\mathrm{Gait}}=4\left(p\right)*1\left(bs\right)*3\left(s\right)*3\left(c\right)*1\left(\mathrm{ax}\right)\\ \;+2\left(p\right)*6\left(bs\right)*3\left(s\right)*3\left(c\right)*1\left(\mathrm{ax}\right)=144. \end{array}$$

$$MNS_{\mathrm{Angle}}=1\left(p\right)*4\left(bs\right)*3\left(s\right)*3\left(c\right)*3\left(\mathrm{ax}\right)=108.$$

$$MNS_{\mathrm{Beta}}=1\left(p\right)*6\left(bs\right)*3\left(s\right)*3\left(c\right)*3\left(\mathrm{ax}\right)=162.$$

$$MNS_{\mathrm{Jerk}}=1\left(p\right)*6\left(bs\right)*3\left(s\right)*3\left(c\right)*3\left(\mathrm{ax}\right)=162.$$

$$MNS_{\mathrm{Entropy}}=1\left(p\right)*6\left(bs\right)*3\left(s\right)*3\left(c\right)*3\left(\mathrm{ax}\right)=162.$$

$$MNS_{RMS}=1\left(p\right)*6\left(bs\right)*3\left(s\right)*3\left(c\right)*1\left(\mathrm{ax}\right)=162.$$

We calculated the propensity to assess percentages using the maximum total number of scores for the 16 subjects as a base.

We used changes in assessment scores to measure subjects’ tendency to change in response to environmental changes. In this study, instead of calculating the evaluated value, we use the number of times the variable changes from ground to height or from height to ground.

Let us look at the total value of the various maximal changes VMC.

We have already calculated the maximal evaluated value of each variable per subject, and based on this, multiplied by the total number of subjects, is the maximal incidence. VMC=16∗MNS .


$$VMC_{\mathrm{Gait}}=\mathrm{NomberSubject}*MNS_{\mathrm{Gait}}=16*144=2304$$

$$VMC_{\mathrm{angle}}=\mathrm{NomberSubject}*MNS_{\mathrm{angle}}=16*108=1728$$

$$VMC_{\mathrm{beta}}=\mathrm{NomberSubject}*MNS_{\mathrm{beta}}=16*162=2592$$

$$VMC_{\mathrm{jerk}}=\mathrm{NomberSubject}*MNS_{\mathrm{jerk}}=16*162=2592$$

$$VMC_{\mathrm{entropy}}=\mathrm{NomberSubject}*MNS_{\mathrm{entropy}}=16*162=2592$$

$$VMC_{RMS}=\mathrm{NomberSubject}*MNS_{RMS}=16*162=2592$$

We computed the score increased or reduced percentage using the following equation.


$$\mathrm{Changed}\;\mathrm{Score}\;\mathrm{Percentage}=\frac{\begin{array}{l} \mathrm{Number}\;\mathrm{of}\;\mathrm{variable}\\ \;\mathrm{score}\;\mathrm{changed} \end{array}}{\;VMC}\;\%$$

## 3 Results

### 3.1 Average modulation of the markers of the perceptual-motor style by height exposure

For each participant within every condition (G1, H, and G2), the aforementioned assessment was conducted, involving the calculation of a total of 100 parameters. The outcomes are presented in Table 1, wherein parameters retaining significance following the adjusted repeated measures analysis of variance (rm-ANOVA) are denoted with a + sign. It is noteworthy that, owing to the application of multiple univariate rm-ANOVAs, a notably low alpha level (Bonferroni correction) was established. Consequently, the presence of the + sign signifies a compelling indication of pronounced change across the conditions.

Table 2 shows the percentage change in each marker of the perceptual-motor style defined in the methods section when the subject walks from ground to height (G1-H), from height to ground (H-G2), and for ground locomotion episodes before and after height exposure (G1-G2).

**Table 2:** Modulation of the markers of the perceptual-motor style by height exposure.

	Gait (%)	Angle (%)	ß factor (%)	Jerk (%)	Entropy (%)	RMS (%)
G1-H	16	5	13	15	7	8
H-G2	25	7	15	19	10	20
G1-G2	25	7	14	17	10	19
Fr	–	6	12	17	11	12
Sg	–	8	16	18	10	18
Tr	–	5	15	16	7	18

The first three rows show the percentages of change in these six markers averaged over the 16 subjects and for the three planes of space (frontal, sagittal, and transverse). Please note one exception: gait markers (length, height, width, duration, step speed, and acceleration) were only calculated in the sagittal plane. The last three rows show the percentage change in score for all subjects in each of the three planes of space (Fr-frontal, Sg-sagittal, and Tr-transverse).

Altogether, Table 2 shows the presence of significant changes in locomotion control in subjects exposed to height, not only while walking at height but also when returning to the ground. On the other hand, there was no significant change in the variables recorded according to the different planes of space, except for entropy. Finally, these averages should not mask the vast heterogeneity of the modulation of locomotion in subjects exposed to height, as detailed below.

### 3.2 Study of the skeletal configuration

**Figure 2:**
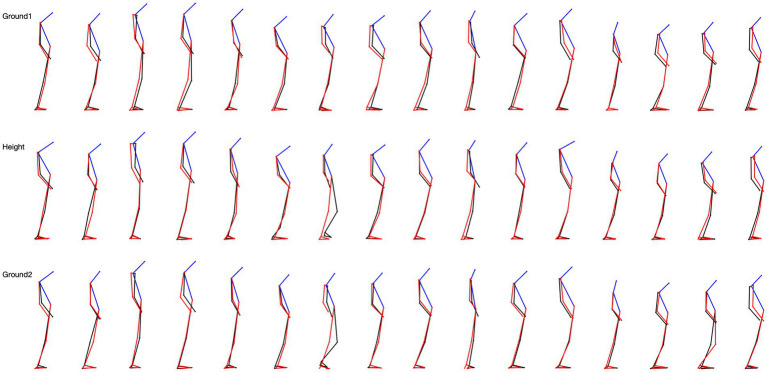
5-step overlapping skeletal posture configuration in sagittal view for 16 subjects. The top row is the situation on the ground, the middle row is the situation at height, and the last row is the situation from height back to the ground.

We studied the skeletal configuration of the participants during locomotion, first on the ground (G1), then at height (H), and again on the ground (G2). That is, we measured the inclination of the head, trunk, forearms, thigh, and leg relative to the gravity vertical in the three planes of space (Figure 2).

#### 3.2.1 In the sagittal plane

- From the ground upwards (G1-H), 52% of the participants modified their posture at the level of the head, little at the upper limbs (8%) and lower limbs (8%), and not at all at the trunk. That is, most participants tilted their heads forward.
- When returning to the ground (H-G2), 56% of participants modified their posture at the level of the head, less at the trunk (20%), and at the upper limbs (20%), and not at all at the lower limbs. Hence, most of the changes were head-straightening.
- There were some changes in the skeletal configuration when comparing the first episode of locomotion on the ground with the second one following the height test (G1-G2). A total of 56% of the participants modified their posture at the level of the head after 10 min at the height. Fewer modifications were observed at the trunk (32%) and upper limbs (32%) levels, and none at the lower limbs level. The changes were head-straightening, and trunk and upper limb positioning varied among persons.

#### 3.2.2 In the frontal plane

- From the ground to height, 24% of the participants changed the posture of the upper limbs, and much less at the head (12%), trunk (12%), and lower limbs (8%). These changes consisted of abduction (24%) of the forearms, a slight inclination of the head in one direction or the other, a straightening of the trunk, and a reduction of the support polygon.
- When returning to the ground, 52% of the participants modified their posture at the upper limbs, less at the lower limbs (20% of participants), at the head (12%), and at the trunk (12%). These changes consisted of a widening of the support polygon and an adduction or abduction of the forearms.
- When comparing the first episode of locomotion on the ground with the second one following the height test, 44% of the participants modified their posture at the upper limbs (abduction or adduction), 24% at the lower limb, and 8% at the head and trunk.

#### 3.2.3 In the transverse plane

- From the ground to the height, 20% of the participants modified their posture at the level of the head and less at the trunk (12%), and upper and lower limbs separately (8%). The rotations could take place to the right or the left.
- When returning to the ground, 24% of the participants modified their posture at the upper limbs, at the level of the head (20%), and much less at the trunk (12%) and lower limbs (12%). The modification consisted of a decrease in the rotation of the head and trunk.
- When comparing the first episode of locomotion on the ground with the second one following the height test, changes in the skeletal configuration were mainly at the upper limbs (32%), and less at the lower limbs (12%), the head, and the trunk (8%).

#### 3.2.4 Summary

A surprising finding was the very large variability of the skeletal configuration we observed in the participants when exposed to height in every experimental condition. It mainly concerned the position of the head in the sagittal plane and the configuration of the forelimbs in the frontal plane.

### 3.3 Study of the modifications of gait characteristics among experimental conditions

When participants changed from walking on the ground to walking at height (G1-H), some characteristics of their steps were modified. It was the case for their step duration (67% of participants), step width (60%), height (27% of participants), and length (33%). The body velocity was changed at the head (60%) and all other body segments (40%). The body acceleration was affected in a decreasing order at the arm (67%), the feet (60%), the trunk (60%), the lower limbs (50%), and the head (33%).

When participants walked again on the ground after having progressed at height (H-G2), some characteristics of their steps were also modified. This was the case for their step duration (47% of participants), step width (53%), height (60% of participants), and length (80%). The body velocity was changed at all body segments in more than 87% of the cases. The body acceleration was affected in a decreasing order at the arm (93%), the trunk (67%), the head (60%), the feet (53%), and the lower limbs (53%).

The characteristics of walking on the ground before and after height exposure (G1-G2) were also modified. This was the case for the step duration (73% of participants), step width (87%), height (67% of participants), and length (87%). The body velocity was changed at all body segments in more than 80% of the cases. The body acceleration was affected in a decreasing order at the arm (87%), the lower limbs (73%), the trunk (67%), the feet (67%), and the head (40%).

In summary, most of the characteristics of the gait were affected in most participants, but here again we observed a large disparity among subjects.

### 3.4 Study of the dynamic characteristics of locomotion

We studied the dynamic characteristics of locomotion first on the ground (G1), then at height (H), and again on the ground (G2). That is, we measured the ß factor, jerk, RMS, and entropy for the head, trunk, forearms, the two segments of the leg, and the foot in the three planes of space (sagittal, frontal, and transversal planes) for each condition (G1, H, and G2). Then we studied whether these variables changed across conditions, that is, when the participants changed from walking on the ground to walking at height (G1-H) and from walking at height to the ground (H-G2), and we compared the dynamic characteristics of locomotion during the first and second episodes of walking on the ground (G1-G2).

Table 3 summarizes our results. It illustrates the percentage of subjects who exhibited statistically significant modifications in the dynamic characteristics of their locomotion and which variables are concerned during the (G1-H) transition, the (H-G2) transition, and the (G1-G2) condition.

**Table 3:** Summary of each variable across the three conditions.

		G1-H						H-G2						G1-G2					
Variable	Plane	Head	Trunk	Arm	Thigh	Calf	Foot	Head	Trunk	Arm	Thigh	Calf	Foot	Head	Trunk	Arm	Thigh	Calf	Foot
ß factor	Fr	18	48	42	48	30	48	24	30	30	48	36	36	24	18	24	30	30	42
	Sg	42	42	42	54	48	12	36	36	48	78	48	54	24	30	60	60	60	42
	Tr	18	36	42	42	42	30	42	54	42	66	48	36	30	30	60	42	54	48
Jerk	Fr	6	24	18	30	42	12	12	24	24	42	48	60	12	12	30	48	48	54
	Sg	6	6	48	30	12	24	42	24	42	30	12	36	30	48	36	30	12	42
	Tr	30	24	48	36	36	60	48	42	84	48	60	36	24	54	60	42	42	48
Entropy	Fr	6	24	18	30	42	12	12	24	24	42	48	60	12	12	30	48	48	54
	Sg	6	6	48	30	12	24	42	24	42	30	12	36	30	48	36	30	12	42
	Tr	6	18	6	18	30	36	6	6	12	24	42	42	12	12	6	18	30	48
RMS	Fr	12	0	12	24	30	36	18	24	30	30	54	78	18	36	42	48	66	66
	Sg	24	24	18	24	24	36	66	66	78	66	78	78	60	60	66	60	60	60
	Tr	24	24	18	24	24	36	66	66	78	66	78	78	60	60	66	60	60	60

### 3.5 Summary

Some characteristics of the change in the dynamics of locomotion emerged. First, it was largely affected among participants across the three conditions. Second, the changes in the dynamics of locomotion followed a gradient, with more modification taking place at the leg level, then at the trunk level, and finally at the head level. Third, the ß factor emerges as a prominent marker of the influence of height on locomotion during the transition from ground to height (G1-H) and from height to ground (H-G2).

### 3.6 Stake percentage of change

**Figure 3:**
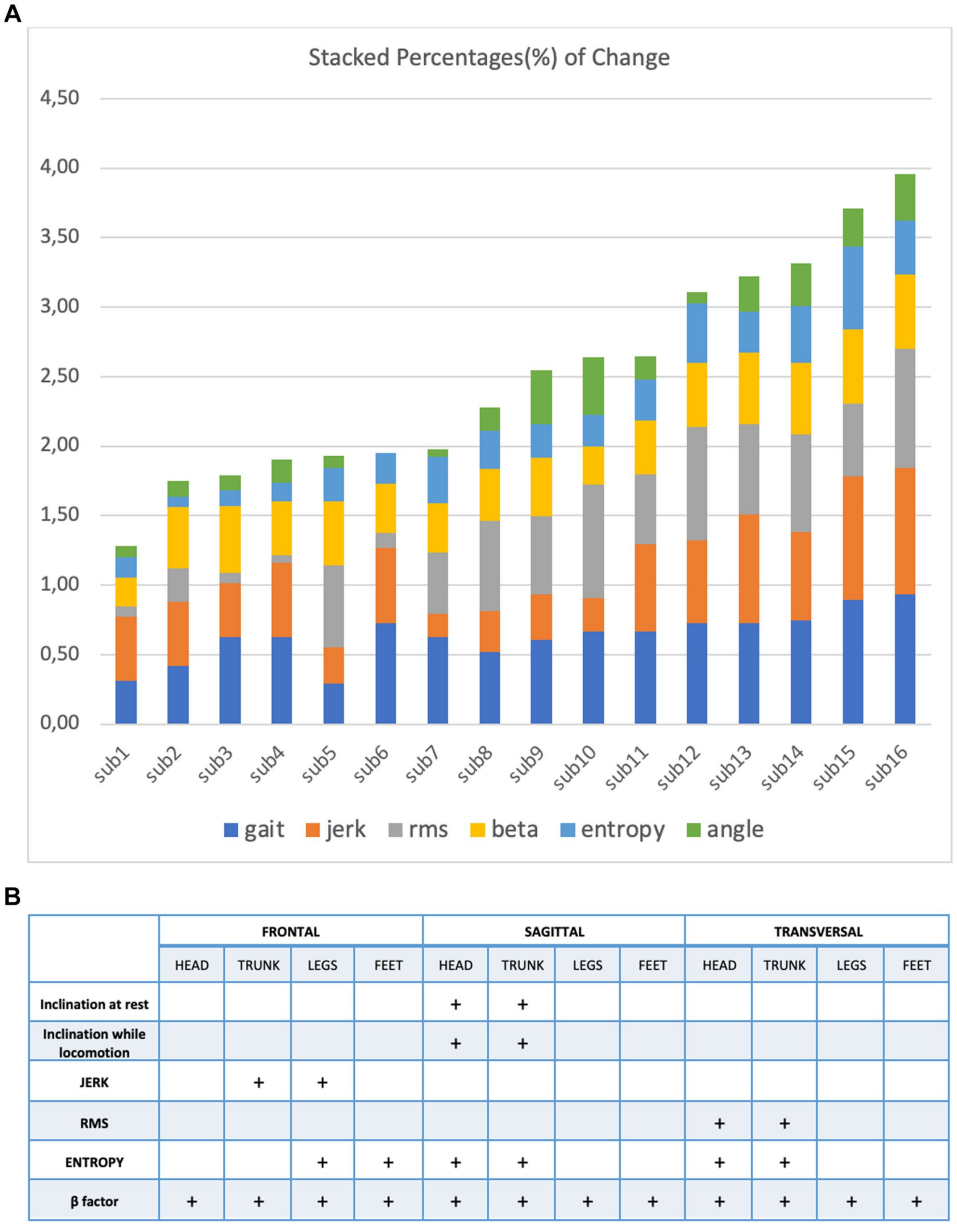
(A) Stacked percentage of change from every variable: gait, jerk, RMS, beta, entropy and angle (B) to characterize the perceptual motor style of individual participants when walking and running on a treadmill at ground level.

Figure 3A illustrates the markers that helped to differentiate the perceptual-motor style of the participants during locomotion at a height. One can make three observations. First, it confirmed that the characteristics of locomotion varied considerably among the 16 subjects exposed to height. Second, their control of locomotion, despite this large variability, varied smoothly; that is, the participants could not be divided into distinct subpopulations. Third, exposure to height affected all variables characterizing locomotion, although their modulation was very different across subjects.

## 4 Discussion

Among the markers we previously used to describe perceptual-motor style during ground locomotion, we identified those that could account for its changes during and immediately after locomotion at height. A surprising result was the great heterogeneity of these changes in the 16 individuals we tested. Static markers (stable head, trunk, and limb configurations relative to the gravitational vertical) and dynamic markers (jerks, entropy, RMS, gate, and two-thirds power law) were modulated by height exposure.

### 4.1 Comparison with previous results

Static balance has been intensively studied during height exposure, but much less so during locomotion (Adkin and Carpenter, 2018). Peterson et al. (2018) studied the effects of VR during beam walking on physiological stress and cognitive load and found no significant difference in walking speed for low and high VR (Parsons et al., 2017). Schniepp et al. (2014), using a 6.7 m-long pressure-sensitive mat, found that when exposed to height, individuals sensitive to visual height intolerance walked more slowly, with reduced cadence, a shorter stride length, and increased double-support phase. Their locomotion was described as a slow, cautious, broad-based gait with small steps. They walked with flat foot contact and less dynamic vertical oscillations of body and head. This gait was not considered specific but rather similar to the cautious gait observed in visually deprived children and adults. Finally, in a study parallel to our own, Zhu et al. (2023) investigated the effects of virtual and physical elevation on physiological stress during height exposure. In this study, plates were placed at the lower ends of the platform, causing slight instability during walking. In addition, a surface layer of foam was introduced to add further postural instability when walking on the raised platform. These instability factors created difficulties that had the effect of increasing the individual’s anxiety level when walking. The platform’s walking space was 2.4 m long and 0.3 m high, while ours was 4 m long and 0.2 m high. Averaging the values recorded in their participants, Zhu et al. (2023) found that increased threat perception (height) prompted the individual to use a cautious walking style with reduced step length and increased steps taken and trial time. Overall, our results are consistent with these earlier findings. They are also consistent with the concept of cautious walking, coined by gerontologists. However, these earlier studies did not aim to detail the motor control of each individual tested at height and therefore did not describe the heterogeneities of their perceptual-motor style.

### 4.2 Exposure to height modulates locomotion. What could be the etiological factors?

As mentioned above, among the markers we previously used to describe perceptual-motor style during ground locomotion, we identified those that could account for its alterations during height locomotion. However, our protocol failed to uncover the causes of the surprisingly large behavioral differences we found between individuals when exposed to height. Presumably, a range of factors were at play, as described by Adkin and Carpenter (2018) in their review.

Fear and anxiety could play a role in modulating the response to height exposure. In the study by Nakahara et al. (2000), recognition that participants were standing at height had two effects: non-acrophobic participants had less body sway at rest if their eyes were uncovered and open, while acrophobic participants showed the opposite response in subsequent trials. In addition to their height intolerance, acrophobic individuals had poorer postural performance in static and dynamic balance tasks (Boffino et al., 2009). Personality traits measured by questionnaires on anxiety and willingness to take physical risks corroborated alterations in static postural control when standing on the edge of a raised platform (Zaback et al., 2019) or during locomotion on the edge of a raised platform (Zhu et al., 2023). In other words, acrophobic people have a fear of falling, and some of the participants we tested exhibited similar symptoms, suggesting that they may share common anxiogenic traits.

The modulation of the perceptual-motor style by exposure to height could also be linked to a greater or lesser visual dependence for the control of balance and locomotion: the distance from the stationary visual scene becomes too great to be detected in order to counteract body movements (Hüweler et al., 2009; Brandt and Huppert, 2014). This leads to impaired motor control, described by Brandt et al. (2015) as a “fear of heights,” resulting in tonic immobility. Fear of falling from a height triggers antigravity muscle co-contractions that increase the sensitivity of sensorimotor balance reflexes. This scenario represents an atavistic motor response resembling death feinting, a primitive behavior widespread in the animal world. The resulting rigid regulation of body sway in turn aggravates the subjective and objective imbalance and initial anxiety, initiating a vicious circle.

Height exposure also modulates postural and locomotor control by influencing how attentional resources are allocated. Several studies have shown changes in attentional focus and alterations in static postural control (see Adkin and Carpenter, 2018 for a review), suggesting that they are causally linked. When threatened, individuals tend to control their posture more consciously (Huffman et al., 2009; Peterson et al., 2018). This may have contributed to changes in some of the markers we used to monitor locomotion at height and, to a variable extent, among the individuals we tested.

Finally, neural networks involved in emotional control have been shown to modulate motor control in animal models, and evidence has also been established in humans. Notably, threat increased muscle spindle sensitivity, ib reflex gain, vestibular control of balance and gaze, and oculomotor control (Adkin and Carpenter, 2018 for a review).

### 4.3 Variability in perceptual-motor style between participants

The high variability we observed among participants for both static and dynamic features of height locomotion and its persistence on the ground is the result of the variability of each perceptual-motor style marker we recorded. This contrasts with the results of our previous study (Vidal and Lacquaniti, 2021) concerning the perceptual-motor style of individuals when walking and running on a treadmill at ground level. These are illustrated in Figure 3B for comparison. In this case, a few markers are enough to differentiate the individuals.

This is reminiscent of a previous study (Bonan et al., 2013) we undertook on control and hemiplegic patients to test their sensitivity to proprioceptive, vestibular, and visual stimuli with regard to postural control (Bonan et al., 2013). Once again, we found that controls and stroke patients showed significant inter-individual variation in response to all three types of sensory stimulation. The control group could be divided into two subgroups according to whether their resting postural control was unaffected or affected by the sensory stimuli. However, none of the hemiparetic patients were insensitive to sensory stimulation. Not only were they excessively dependent on visual information to control their posture, but they were also more sensitive to vestibular and proprioceptive information than the controls. Overall, for both static postural control and locomotion, modulation of perceptual-motor style seems relatively straightforward to characterize in healthy subjects, both in terms of markers and taxonomy. In contrast, when pathologies occur or challenging conditions are tested, the perceptual-motor styles become more heterogeneous and affect more markers of motor control.

### 4.4 A gradient in terms of timing and variability during locomotion at a height

In a previous study, we tested subjects with multiple balance perturbations provided by unpredictable translations of the supporting surface in different directions and speeds (Le Goic et al., 2018). Our data showed that there is little time to adjust the way one falls from a standing position. During the initial part of a fall, the observed trajectory results from the interaction between the destabilizing external force and the body; the intrinsic inertial properties of joints, ligaments, and the musculotendinous system then have a major contribution. This passive phase is then followed by an active phase, which consists of a corrective response to the postural perturbation. The motor synergies at play followed a temporal gradient from the limb to the neck muscles. Furthermore, our results revealed that visual and vestibular information could not detect the fall at its onset because the head remained stable with respect to space. That is the participants are prepared for the impact on the basis of the proprioceptive information. Similarly, in the present study, the variability of the movements of the body segments during the locomotion on the plank followed an ascending order from legs to head. In order to control the cautious gait on the plank, visual information was also of no use, being the distance to the ground; vestibular information contributed little, being the low-frequency content of the head movement. That is, proprioceptive afferences were also the main source of information to control locomotion. Therefore, it would be the emphasis on proprioceptive information during fall and the cautious gait that would explain the leg-to-arms-to gradient we observed both in terms of timing and variability.

### 4.5 Limitations

Our study had several limitations. Our sample size was small. Although the participants were of approximately the same age and cultural background and had no pathology, a larger panel might have revealed an age dependency and/or established a partition in the three types of populations, as illustrated in Figure 3A. Furthermore, we did not record participants’ visual dependence, neurovegetative responses, or attention allocation strategies during postural and locomotor control, nor did we test their psychological traits using suitable questionnaires. This could have explained the underlying causes of heterogeneity in participants’ perceptual-motor style when exposed to height. Another limitation of this study was the extensive multiple testing procedures and the application of the Bonferroni correction of *p*-values, which, while controlling for Type I errors, may have increased the likelihood of Type II errors by overly conservative adjustment, potentially leading to the non-detection of true effects. Nevertheless, this limitation can be interpreted as substantiating the robustness of the results presented in this study. It suggests the possibility that less pronounced changes, yet to be identified, may exist and warrant investigation in future research.

## Data availability statement

The raw data supporting the conclusions of this article will be made available by the authors, without undue reservation.

## Ethics statement

The studies involving humans were approved by CER U-Paris N° 2021-12-WANGVIDAL Présidente: Jacqueline Fagard. PROTOCOLE: Influence of fear stimuli under virtual reality to the posture control. Noms du/des chercheur(s): DW and P-PV. Email pour la correspondance: danping.wang@parisdescartes.fr; pierre-paul.vidal@parisdescartes.fr. Labo/Service: Plateforme d’Etude de la Sensorimotricité; Université de Paris, Évalué à la séance du 6 avril 2021. AVIS: favorable. The studies were conducted in accordance with the local legislation and institutional requirements. The participants provided their written informed consent to participate in this study. Written informed consent was obtained from the individual(s) for the publication of any potentially identifiable images or data included in this article.

## Author contributions

DW: experience, signal processing, data analysis, paper writing. IB and NV: data analysis. JC and LO: signal processing. P-PV: data analysis and paper writing. All authors contributed to the article and approved the submitted version.

## Funding

National Key Research and Development Program of China under Grant 2021YFE0100100.
